# Lipid Changes 10–15 Years After Roux-en-Y Gastric Bypass: A Prospective Observational Study

**DOI:** 10.1007/s11695-024-07601-x

**Published:** 2025-01-15

**Authors:** Eirin Rosø Barkhall, Johanne Tro, Jorunn Sandvik, Siren Nymo, Bård Kulseng, Gjermund Johnsen, Dag Arne Lihaug Hoff, Torstein Hole

**Affiliations:** 1 Department of Clinical and Molecular Medicine, Faculty of Medicine and Health Sciences, Norwegian University of Science and Technology (NTNU), 7491 Trondheim, Norway; 2 Obesity Research Group, Department of Clinical and Molecular Medicine, Faculty of Medicine and Health Sciences, Norwegian University of Science and Technology (NTNU), 7491 Trondheim, Norway; 3 Centre for Obesity, Clinic of Surgery, St. Olav’s University Hospital, 7006 Trondheim, Norway; 4 Department of Surgery, Møre Og Romsdal Hospital Trust, 6026 Ålesund, Norway; 5 Clinic of Surgery, Namsos Hospital, Nord-Trøndelag Hospital Trust, 7601 Levanger, Norway; 6 National Advisory Unit On Advanced Laparoscopic Surgery, St. Olavs University Hospital, 7006 Trondheim, Norway; 7 Department of Clinical Studies, Møre Og Romsdal Hospital Trust, Ålesund, Norway; 8 Department of Health Science Ålesund, Faculty of Medicine and Health Sciences, Norwegian University of Science and Technology (NTNU), 7491 Trondheim, Norway; 9 Medical Department, Ålesund Hospital, Møre Og Romsdal Hospital Trust, 6026 Ålesund, Norway; 10 Faculty of Medicine and Health Sciences, Norwegian University of Science and Technology (NTNU), 7491 Trondheim, Norway

**Keywords:** Lipid profile, Gastric bypass, BMI, Weight loss

## Abstract

**Background:**

Several studies have documented a beneficial short-term effect on lipid profile after Roux-en-Y gastric bypass (RYGB), but there is limited data on long-term changes.

**Objectives:**

To describe long-term (> 10 years) changes in lipid profile after RYGB and to explore the relationship of lipid changes to changes in weight and baseline and demographic parameters.

**Methods:**

The BAROBS study is a prospective observational study post RYGB conducted at three different hospitals. Surgical procedures were performed between 2003 and 2009, and the collection of data was in 2018–2020. Data on lipid profile, weight, body mass index (BMI), percentage of total weight loss (%TWL), and pre- and postoperative type II diabetes mellitus (DMII) was collected at baseline, 1–2, 5, and 10 years post-surgery and was available for 314 of 546 patients in the study.

**Results:**

The mean (SD) follow-up was 11.5(± 1.5) years, with a mean reduction of 11.7% for LDL, 29.7% for TG, and 7.7% for total cholesterol compared to baseline. Except for HDL and total-/HDL-cholesterol-ratio, all lipid variables reached their greatest change after 1–2 years with an attenuation of changes at end of study. HDL and total-/HDL-cholesterol-ratio had stable values from 5 to 10 years post-surgery. Lipid profile improved more in patients with greater weight loss. There was a 59% reduction in DMII at end of study, and there was a significant relation between preoperative DMII and long-term lipid values.

**Conclusion:**

There is an improvement of all lipid parameters after 10 years post RYGB related to both the magnitude of weight loss and the presence of DMII.

**Graphical Abstract:**

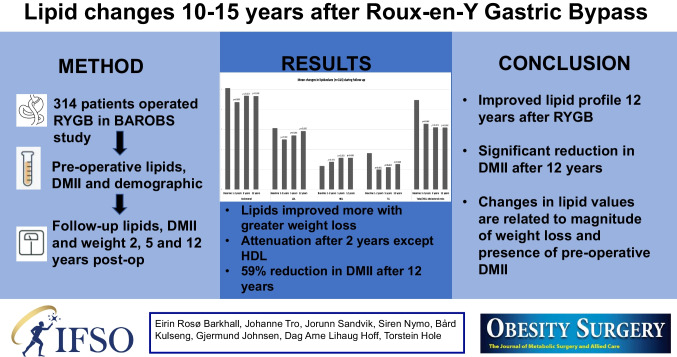

## Introduction

There is a relationship of overweight (BMI > 25 kg/m^2^) and obesity (BMI > 30 kg/m^2^) to an increased risk of cardiovascular disease (CVD) and vulnerability for developing a series of conditions such as dyslipidemia, hypertension, obstructive sleep apnea, type II diabetes mellitus (DMII), and several types of cancer [1, 2].

Dyslipidemia is a well-known risk factor for developing CVD independent of BMI [1, 3]. The majority of patients with severe obesity corresponding to BMI > 40 kg/m^2^ will present lipid and lipoprotein abnormalities characterized by both increased fasting and postprandial triglyceride (TG) levels, low levels of high-density lipoprotein cholesterol (HDL), and increased low-density lipoprotein cholesterol (LDL) [2, 4–8].

Today, the most common bariatric surgery procedures are laparoscopic sleeve gastrectomy (LSG) and laparoscopic Roux-en-Y gastric bypass (RYBG). Surgery is optioned at BMI > 40 or > 35 with obesity-related illness. Both procedures have proven to have positive effects on dyslipidemia and other obesity-related conditions such as DMII [9–14]. Bariatric surgery consistently improves lipid profile and reduces cardiovascular risk independent of preoperative cardiovascular disease state [12, 15]. However, several studies suggest that RYGB gives a greater change in lipid profile, especially regarding LDL and total cholesterol [16–19].

The majority of studies on lipid changes had a maximum follow-up time of approximately 5 years and described changes to occur already in the early postoperative period, even before weight reduction [7]. The changes then reach a peak after 1–3 years before lipid changes are attenuated the following years [12, 19, 20]. However, still with an overall improved lipid profile after 5 years when comparing to preoperative values [8, 12, 16, 17, 19].

To our knowledge, there exist only six studies on lipid profile post RYGB with a follow-up for 10 years or more, published between 2016 and 2024 [12, 20–24]. These studies found a sustained improvement of dyslipidemia 10 years post-surgery. Gero et al. found a correlation between weight loss and changes in lipid values, with greater improvement in patients with percent total weight loss (%TWL) above 25 or percent excess body mass index loss (%EBMIL) above 50 when comparing 5-year results to preoperative values [12]. There is, however, a lack of data on the relationship of changes in lipid profile and preoperative parameters and changes in BMI or weight 10 years or more after surgery.

The aim of this study was thus to describe the long-term changes (> 10 years) in lipid profile after RYGB and to explore if there is any relationship between lipid changes and weight changes and other selected baseline and demographic parameters.

## Methods

### Population

The BAROBS study is a prospective observational study of patients who had follow-up > 10 years after RYGB. This is a sub-study of the BAROBS study of 959 patients who underwent RYGB between 2003 and 2009 at three hospitals in the region of Mid-Norway. To be offered RYGB, patients had to be aged between 18 and 60 years with a BMI > 40 kg/m^2^ or BMI > 35 kg/m^2^ with obesity-related comorbidity, according to the national guidelines at the time of the procedure [25, 26].

**Fig. 1:**
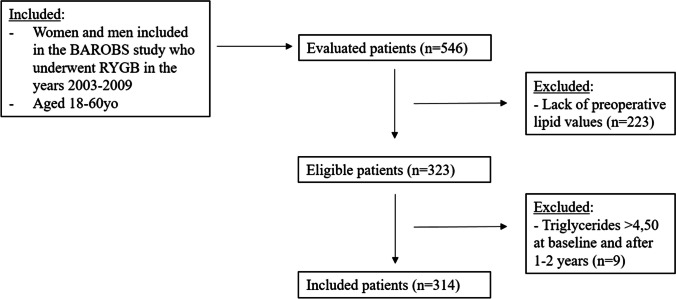
Study population

Twenty-nine patients died during the observation period. The remaining 930 patients were eligible for inclusion in the BAROBS study, of which 546 patients attended their follow-up between August 2018 and June 2020 and thus were included in the BAROBS study. Of these, 323 had registered lipid values at baseline and were eligible for this sub-study. The LDL levels at baseline and 1–2 years postoperatively were calculated using the Friedewald formula, and patients with TG levels > 4.50 mmol/L were excluded (*n* = 9), and 314 patients were thus included in the study (Fig. 1).

The number of patients treated for hyperlipidemia pharmaceutically was almost concurrent at baseline and end of study (21 to 25 patients) and is not excluded from the study.

### Biochemical Assessments

A set of blood tests with all lipid parameters, including s-cholesterol, s-LDL, s-HDL, s-TG, and weight with calculation of BMI, %TWL, as well as presence of DMII, were done at baseline, 1–2, 5, and 10 years after surgery.

For biochemical analyses of lipid variables, fasting venous blood was analyzed by standard accredited methods. The hospitals used slightly different methods and equipment at different times, but the variations between methods were limited and mainly underestimated the differences at end of study.

Direct measurement of LDL was implied at different times at the respective hospitals. For values at baseline and after 1–2 years, LDL was calculated using the Friedewald formula. A few LDL values from the period were available through data collected by general practitioners. The estimated values were compared to the few LDL levels registered at baseline and after 1–2 years and had high agreement with the registered.

There were only a few missing single lipid values mainly regarding triglycerides and LDL (*n* = 1–15), but data was almost complete at baseline and end of study (ES) for all participants. Total-/HDL-cholesterol ratio was calculated for comparison between baseline and ES.

### Guidelines for Dyslipidemia

European Society of Cardiology (ESC) recommends LDL < 2.6 mmol/L for otherwise healthy individuals, and Norwegian guidelines recommend TG < 1.7 mmol/L along with HDL > 1.0 mmol/L for men and > 1.2 mmol/L for women. Different recommendations for both guidelines apply for patients with comorbidities. In Norway, the NORRISK2 is recommended for clinical risk calculation for individual patients > 45 years of age. All elements in the risk calculator were not available, and thus, recommended thresholds for individual lipid values were used in our calculations [1, 27, 28].

### Statistical Methods

Normally distributed variables are reported as mean ± standard deviation (SD) along with confidence intervals (CI). QQ-plot was performed to check for the normal distribution of continuous variables. Student’s *T*-test was used to compare means for normally distributed parameters. For non-normal distributed variables, adequate non-parametric tests were performed (McNemar Change Test).

To evaluate any relationships between absolute lipid values at end of study as well as changes in lipid values from baseline to end of study, with baseline parameters and changes in BMI (%TWL), multiple linear regression analyses were performed. Backward model with variables with *p*-values < 0.2 in univariate analyses included in the model.

The number of patients who exceeded guidelines for recommended TG and HDL adjusted for sex, or recommended levels for LDL, was calculated. Baseline and end of study population fractions were compared [1, 28].

Two-sided *p*-values < 0.05 were considered significant. No corrections regarding multiple testing were done. The statistical analyses were performed using IBM SPSS version 28.0.1.0 (SPSS Inc. Chicago, IL, USA).

### Ethics

The study was approved by the Regional Committee for Medical and Health Research Ethics (2017/1828 REK South-East Norway), and all patients gave informed consent. All procedures performed in the study were in accordance with the ethical standards of the National Research Committee and with the 1964 Helsinki Declaration and its later amendments.

## Results

Among the 314 patients included in this study, there was a mean follow-up time for 11.5 (range 9-16.5) years. There was a preponderance of women compared to men, and most of the patients included were in the age group 30-39 years (Table 1). End of observation period complete lipid profile was available for 99.7% of participants. Fifty-one (16.2%) patients had DMII at baseline and 21 (6.7%) at end of observation period (*p* < 0.001). Changes in BMI, %TWL, DMII, hyperlipidemia, and lipid values are presented in Table 2.

**Table 1:** Distribution of age and sex in numbers and percentage at baseline

Age	Total, mean (15)	Women, mean (15)	Men, mean (15)
	39.5 ± 8.7	39.3 ± 8.5	40.6 ± 8.8
	Groups	Numbers (***n*** = 314)	Percentage
	19–29	37	11.8%
	30–39	130	41.4%
	40–49	99	31.5%
	50–59	48	15.3%
Sex	Women	257	81.8%
	Men	57	18.2%

**Table 2:** Lipid values and weight variables at baseline, 1–2 years, 5 years, and at end of observation period (EO)

Variables	Baseline	1–2 years	5 years	EO	p*	CI
BMI (kg/m^2^), mean (SD)	44.2 ± 5.7	29.3 ± 4.9	32.2 ± 6.3	35.0 ± 7.0	< 0.001	8.59–9.89
%TWL*,* mean (SD)	-	33.5 ± 8.80	27.0 ± 10.9	21.2 ± 12.5	< 0.001	11.15–13.55
Total cholesterol (mmol/L), mean (SD)	5.07 ± 0.96	4.40 ± 0.79	4.69 ± .080	4.68 ± 0.82	< 0.001	0.30–0.50
LDL (mmol/L), mean (SD)	3.07 ± 0.83	2.51 ± 0.68	2.71 ± 0.71	2.94 ± 0.76	0.006	0.04–0.22
HDL (mmol/L), mean (SD)	1.18 ± 0.26	1.38 ± 0.31	1.58 ± 0.38	1.58 ± 0.41	< 0.001	− 0.44 to − 0.37
Triglycerides (mmol/L), mean (SD)	1.82 ± 0.74	1.00 ± 0.46	1.11 ± 0.53	1.28 ± 0.52	< 0.001	0.46–0.62
Total/HDL-cholesterol ratio, mean (SD)	4.48 ± 1.17	3.29 ± 0.79	3.11 ± 0.85	3.10 ± .079	< 0.001	1.26–1.49
Pharmaceutical cholesterol treatment (*n*, %)	*n* = 21 (6.7%)			*n* = 25 (7.96%)	0.541	
Type II diabetes (*n*, %)	*n* = 51 (16.2%)			*n* = 21 (6.7%)	< 0.001	
Insulin	*n* = 12			*n* = 5		
Per oral pharmaceuticals	*n* = 32			*n* = 20		
Diet regulated	*n* = 7			*n* = 0		

*EO* end of observation period (10 years or more post-surgery)

^*^For variables after 10 years or more compared to baseline

**Fig. 2:**
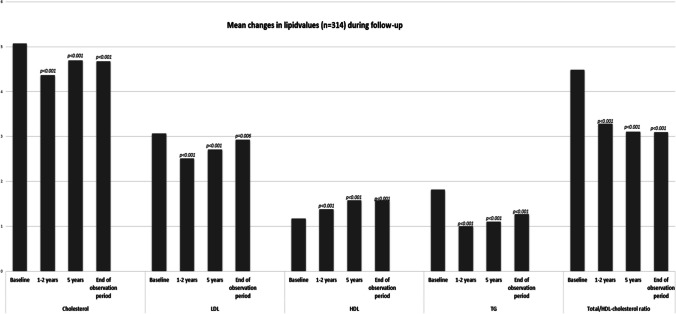
Mean changes in lipid variables during follow-up. *p*-values are relative to baseline values

**Fig. 3:**
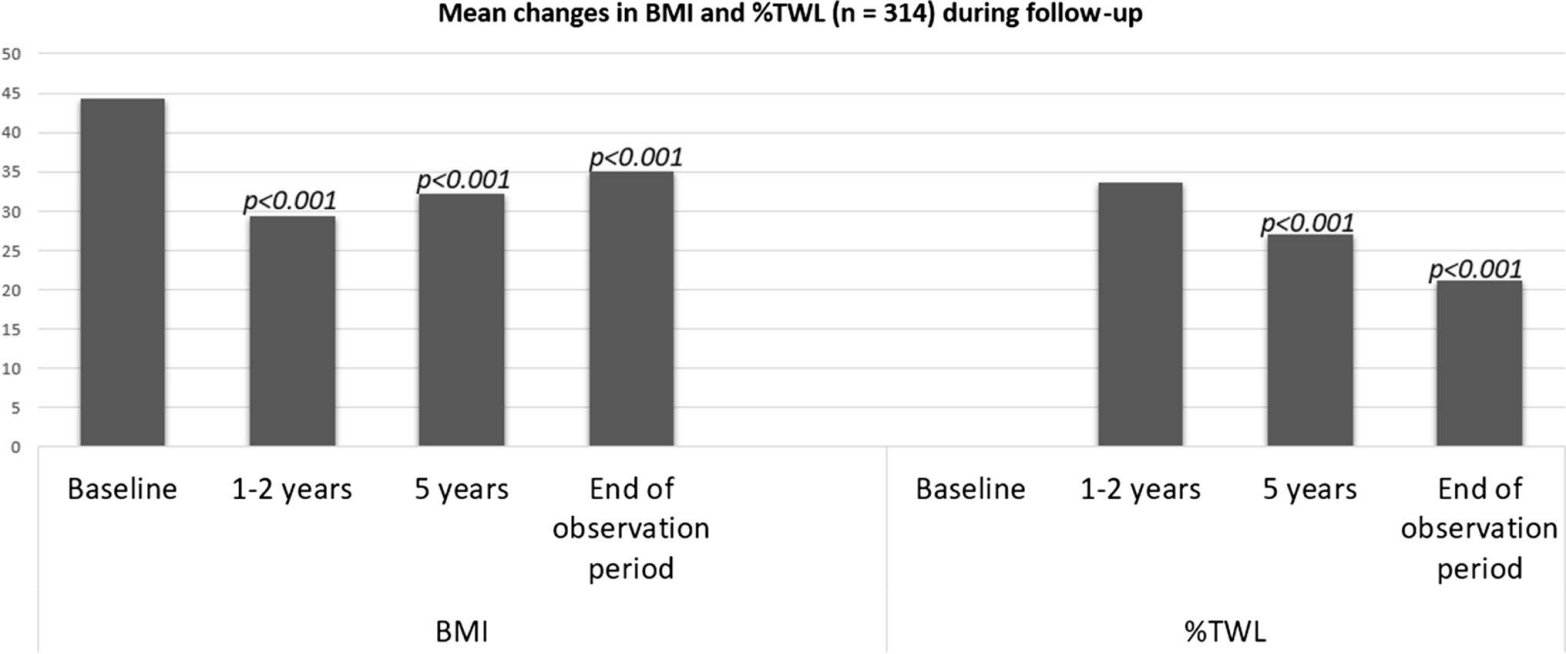
Mean changes in BMI and %TWL during follow-up. *p*-values are relative to baseline values

All lipid variables were significantly improved at end of observation period (Table 2, Fig. 2). Except for HDL and total-/HDL-cholesterol ratio, improvements peaked at 1–2 years post-surgery with an attenuation of the changes at 12 years (Fig. 2). This reflects the same pattern as seen in weight changes during the time of follow-up (Fig. 3). HDL and total-/HDL-cholesterol ratio had stable values from 5 to 10 years post-surgery (Fig. 2, Table 2).

Two hundred and twenty-six (72%) patients had LDL levels > 2.6 mmol/L at baseline, and 198 (63%) after 12 years. This gives a 12.4% reduction in patients who did not reach ESC guidelines (1). Applying Norwegian guidelines based on combined values for HDL and TG, 91 patients (29%) had increased levels at baseline and 17 patients (5.4%) after 12 years (28).

**Fig. 4:**
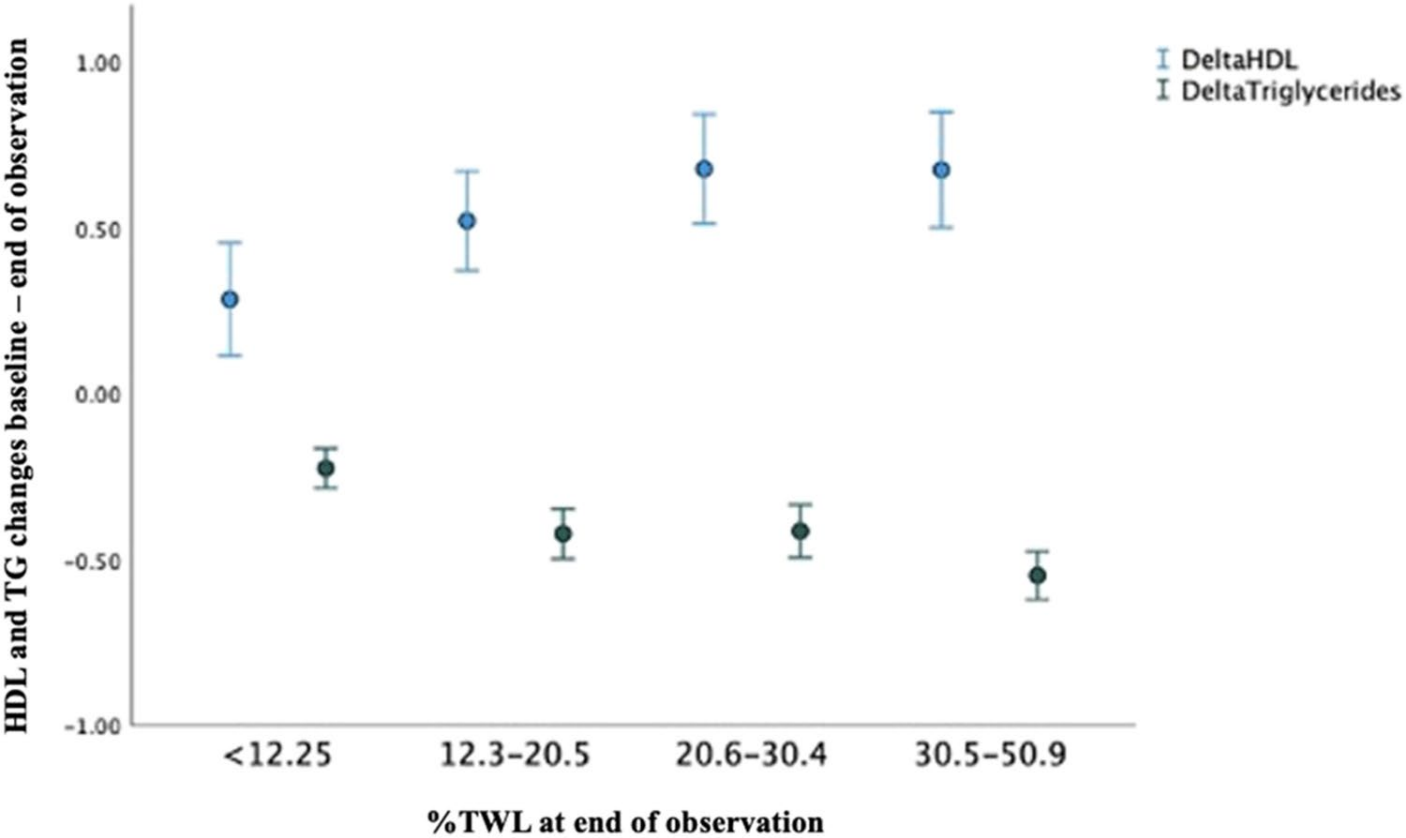
HDL and TG changes in relation to %TWL quartiles at end of observation period

The relationship of absolute lipid values at the end of the observation period and changes (Δ) from baseline compared to BMI and other parameters are presented in Table 3. Percentage of total weight loss (%TWL) at end of observation period showed a relation with Δ-TG and Δ-HDL (Fig. 4). As the figure illustrates, the greater the %TWL, the greater the effect on Δ-TG and Δ-HDL. For Δ-LDL, BMI at 12 years was the only significant factor, and for Δ-cholesterol, it was %TWL after 1–2 years. Thus, weight changes correlated with all relative changes in lipid values.

**Table 3:** Predictors of absolute lipid values and changes in lipid values from baseline to end of observation period

Parameters	Total cholesterol		LDL		Triglyceride		HDL	
	*R*^2^	*p*	*R*^2^	*p*	*R*^2^	*p*	*R*^2^	*p*
Lipid values at end of observation period								
Age at end of observation	0.079	< 0.001	0.007	0.027	0.171	0.010	-	-
Sex	0.079	0.011	-	-	-	-	0.236	< 0.001
BMI baseline	-	-	-	-	0.171	< 0.001	0.236	< 0.001
BMI after 1–2 years	-	-	-	-	-	-	-	-
BMI at end of observation	-	-	-	-	-	-	-	-
%TWL after 1–2 years	-	-	-	-	-	-	-	-
%TWL at end of observation	-	-	-	-	0.171	< 0.001	0.236	< 0.001
Preoperative DMII	0.079	0.001	-	-	0.171	0.002	0.236	0.005
DMII at end of observation	-	-	-	-	-	-	-	-
Changes in lipid values from baseline to end of observation period								
Age at end of observation	-	-	-	-	-	-	-	-
Sex	-	-	-	-	0.073	0.043	0.223	< 0.001
BMI baseline	-	-	-	-	-	-	0.223	< 0.001
BMI after 1–2 years	-	-	-	-	-	-	-	-
BMI at end of observation	-	-	0.055	< 0.001	-	-	-	-
%TWL after 1–2 years	0.013	0.04	-	-	-	-	-	-
%TWL at end of observation	-	-	-	-	0.073	< 0.001	0.223	< 0.001
Preoperative DMII	-	-	-	-	0.073	0.032	0.223	0.012
DMII at end of observation	-	-	-	-	-	-	-	-

Preoperative DMII proved a significant predictor regarding HDL, TG, and total cholesterol after 12 years. Age was significantly related to absolute values for TG, LDL, and total cholesterol, while sex was significant for absolute values for HDL and total cholesterol, as well as for Δ-HDL and Δ-TG (Table 3).

## Discussion

The main findings of this study are a significant improvement in all lipid variables in the entire follow-up period (> 10 years), with the attenuation of the changes from 1 to 2 years post-surgery to end of observation period except for HDL and total-/HDL-cholesterol-ratio which has stable values from 5 to 10 years. Long-term weight loss was substantial, and lipid profile improved more in patients with greater weight loss. Additionally, patients who had DMII preoperatively had a greater improvement in overall changes in lipid values at 12 years. This relation has not previously been described in a long-term perspective.

In accordance with results from other long-term studies, a statistically significant improvement on weight and lipid levels was documented [12, 20–23]. Three studies have previously described a peak in both lipid changes and weight loss after 2 years, similar to nadir found in this study [12, 19, 20]. LDL, total cholesterol, and TG show a continuous attenuation towards baseline levels but still have an overall improved profile at 12 years, with 1/4 of the initial (nadir) effect sustained for LDL, 1/2 for total cholesterol, and 2/3 for TG. The relation between weight/BMI and lipid variables complies with previous research with follow-up for up to 5 years [12]. Weight loss followed the same development as lipid changes. At 12 years, %TWL was 21.2%, which corresponds with findings in other studies [12, 20, 21].

When exploring relations to other parameters, BMI and %TWL showed significant relationship to end of observation lipid levels. For changes in TG and HDL, %TWL at end of follow-up proved significant, whilst BMI did not. This might imply percentage of body mass lost is more significant than the exact BMI at the end of the follow-up. To our knowledge, none of the long-term studies (> 10 years) has examined the relationship between changes in lipid levels at the end of follow-up with other parameters. Gero et al. point out the coherence between weight loss and lipid improvement after 5 years, with a greater improvement in patients with greater weight loss [12]. These findings are extended with our study with an even longer observation period. While this study explores the relation between selected parameters and lipid values, increasing age during the follow-up period can also partially explain the gradual increase in lipid levels post nadir.

HDL and total-/HDL-cholesterol ratio changes did not follow weight development the same way as remaining lipid variables, presenting stable values from 5 to 10 years post-surgery (Figs. 2 and 3). This finding is supported by previous research [12, 20]. HDL is influenced by other factors such as physical activity and a healthier diet, which might be reasonable to assume for bariatric surgery patients [29].

Compared to guideline thresholds, there was a 12% and 81% reduction from baseline to ES study in patients with lipid levels who did not meet ESC and Norwegian guidelines. These improvements are supported by other long-term studies where dyslipidemia prevalence decreased by 46% and 52.4% 10 years after RYGB [12, 21]. The total-/HDL-cholesterol ratio improved from 4.48 (± 1.17) at baseline to 3.10 (± 0.78) at end of study (*p* < 0.001; Table 2), also supporting an improved CVD risk profile (Fig. 2).

For patients with preoperative DMII, there was a greater enhancement of absolute values for HDL, total cholesterol, and TG; this relation has to our knowledge never been described in a 10-year perspective. During this study, there was considerable improvement with a 59% decrease in DMII from baseline to ES. Other studies with 10–12-year follow-up time have found similar results with 51% and 54.2% remission rates [21, 23]. Kim et al. argue that DMII’s relation with metabolic syndrome can explain how this patient group can have different premises for abnormalities in lipid profile [30]. DMII remission can occur in response to relative weight loss and give changes in metabolism in patients who remain overweight. This might provide an explanation on how DMII remission can be an influential factor on end of follow-up lipid variables.

### Strengths and Limitations

The BAROBS data consists of a patient cohort with a long observation time with nearly no loss to follow-up throughout the period, and information on the use of lipid-lowering drugs both at baseline and at the end of follow-up was available.

The greatest weakness of the study is the various use of methods for analyzing lipid values during the study period. However, an analysis between methods was performed, and the overall conclusion was that these methodological changes underestimated the true changes of LDL and HDL. For TG, there was both a slight overestimation and underestimation at different hospitals, and for total cholesterol, there was next to no divergence between methods. Thus, for all practical purposes, this has decreased the positive changes for LDL and HDL, and there should be a minor risk of overestimating results due to bias in the analytical methods used during the observation period.

Seventy-two percent of patients had LDL levels > 2.6 mmol/L at baseline, and 28% had above 3.5 mmol/L, but we have not performed any sub-group analyses to explore if there is any difference in response between patients with and without hyperlipidemia.

This study has limited information about other factors which also can influence lipid variables, such as lifestyle changes or other baseline values than those registered in the BAROBS study.

Finally, this is an observational study, with the limitations and weaknesses of this design.

## Conclusion

This study gives a unique insight into long-term changes in lipid values post RYGB surgery and substantiates previous research with a significant improvement of all lipid parameters after 10 years. In addition, the study provides new information on relations between preoperative DMII, weight loss, and long-term changes in lipid composition and levels. These relations have not previously been described in relation to long-term follow-up after RYGB. These relationships should be examined further with studies including different geographic regions and ethnicities.
